# Callispheres drug-eluting bead transhepatic artery chemoembolization with oral delivery of sorafenib for the treatment of unresectable liver cancer

**DOI:** 10.3389/fsurg.2022.981116

**Published:** 2022-09-02

**Authors:** Wenhui Wang, Fenqiang Li, Peiying Gan, Baohua Li, Shuangxi Li

**Affiliations:** ^1^ Department of Interventional Medicine, The First Hospital of Lanzhou University, Lanzhou, China; ^2^ Department of Neurosurgery, The First Hospital of Lanzhou University, Lanzhou, China

**Keywords:** drug-eluting bead, Callisphere, transarterial chemoembolization, sorafenib, liver cancer

## Abstract

**Objective:**

Liver cancer is a significant contributor to global burden of cancer. Transcatheter arterial chemoembolization (TACE) is the standard of care for patients with unresectable liver cancer, and CalliSpheres, as novel drug-eluting bead (DEB) microspheres, have been found to be associated with a high tumor response rate. However, the outcomes after DEB-TACE treatment are not always satisfactory with tumor recurrence. Herein, we attempt to compare the clinical efficacy and safety of DEB-TACE with sorafenib and conventional TACE in treating advanced liver cancer.

**Methods:**

The study retrospectively reviewed clinical records of 96 patients with liver cancer, among which there were 48 cases receiving DEB-TACE with sorafenib and 48 cases receiving conventional TACE. The physical properties of Callispheres were evaluated in HepG2 cells and a B6/J mouse model.

**Results:**

DEB-TACE with Callispheres were demonstrated to effectively maintain stability and prolong the half-life of epirubicin. Compared with the patients receiving conventional TACE, those receiving DEB-TACE with sorafenib exhibited better patient outcomes with increased survival rate, reduced tumor volume, and declined levels of tumor markers. Additionally, DEB-TACE with Callispheres could effectively protect liver function, as well as reduce the toxic effects of loaded epirubicin, and its combination with sorafenib would not increase the incidence of adverse reactions.

**Conclusion:**

DEB-TACE using CalliSpheres combined with sorafenib could prevent the progression of liver cancer and bring a better prognosis.

## Introduction

According to the WHO/IARC for cancer statistics (year 2020, worldwide, both sexes, all ages) liver cancer had a higher crude rate of incidence (11.6%) and mortality (10.7%) (1). It was accepted as one of the digestive tumors in China with the estimated number of new cases and deaths of 410, 038 and 391, 152 (2). Because the onset of liver cancer is hidden, and early symptoms are not obvious, patients have often reached the middle and late stage when diagnosed. At this time, liver cancer progresses rapidly, and related complications have often occurred, so patients have already missed the best opportunity for surgical treatment (3). Otherwise, because of the relative shortage of liver donors in China, the vast majority of patients cannot get curative treatment through surgery. At present, for patients with advanced liver cancer, the conventional treatment is mainly radiotherapy and chemotherapy, but due to the more adverse reactions and poor treatment pertinence, the clinical treatment is often not good (4).

The invasive growth of tumor cells is closely related to the supply of blood nutrients, as a result, if the blood supply of liver tumors can be selectively blocked, it will greatly improve the pertinence and clinical efficacy of treatment, and create surgical opportunities for advanced patients (5). Liver blood supply mostly comes from portal vein, and the rest from hepatic artery, on the contrary, the liver cancer blood supply almost all comes from hepatic artery (6). As the different blood supply modes of normal liver tissue and liver cancer, and with the continuous progress of medical technology, transcatheter arterial chemoembolization (TACE) can be implemented in the treatment of hepatocellular carcinoma and has been accepted by more and more clinicians (7). The embolic agent used in conventional TACE (c-TACE) is mainly a mixture of iodized oil and chemotherapeutic drugs. The mixture blocks the target blood vessels through the siphon principle, thus blocking the blood supply of liver cancer, and releases chemotherapeutic drugs around tumor cells at the same time (8). However, excessive iodized oil will cause irreversible damage to the liver, and some scholars have found that iodized oil will be decomposed by tumor cells, and finally degraded by monocyte macrophage system, resulting in the recanalization of tumor blood supply (9). In addition, due to the instability of the mixture, chemotherapeutic drugs are easy to leave the target and enter the peripheral blood circulation, resulting in the decrease of drug concentration at the tumor target and the increase of drug concentration in peripheral blood (10, 11), which aggravates the adverse reactions of drugs. Therefore, the c-TACE scheme needs to be improved to better block the blood supply and stably release chemotherapeutic drugs.

With the continuous progress of medical technology, the improved scheme of drug loaded microspheres for chemoembolization has been proposed. Among them, Callispheres DEB is the latest approved in China (12). Callispheres DEB is mainly synthesized from polyvinyl alcohol and has good biocompatibility with the body. It can choose different diameters according to the different target segments of the blocked hepatic artery, so as to better block the target vessels (13). In addition, Callispheres DEB has good compliance, and its size can be compressed by 50% at most in interventional therapy (14), so that it is not easy to block the microcatheter. When Callispheres DEB reaches the target blood vessel, it can quickly restore the original size and achieve the effect of accurately cutting off the blood supply of the tumor. Moreover, Callispheres DEB cannot be degraded *in vivo*, so that it can permanently cut off the blood supply of the tumor and reduce the number of operations (15). Compared with the traditional unstable mixture, Callispheres DEB have better stability by loading chemotherapy drugs through ion bonds. After reaching the action target, Callispheres DEB can slowly release chemotherapy drugs by exchanging with sodium particles in the blood (16). As a result, it can avoid too many chemotherapy drugs from entering the peripheral blood, on the other hand, it can ensure effective and continuous drug concentration around tumor cells and reduce the dosage of drugs (17), so as to improve the curative effect and reduce the toxic and side effects of chemotherapy drugs.

As a multi kinase inhibitor, sorafenib was the first targeted therapy approved for advanced renal cell carcinoma, transforming treatment, which was reported to effectively reduce angiogenesis, thus reducing blood supply of liver cancer cells with a well characterized tolerability and safety profile (18, 19). However, there are few studies on the treatment of hepatocellular carcinoma with Callispheres DEB TACE combined with sorafenib. This study discusses the therapeutic effect of combined therapy in advanced liver cancer, in order to find a better clinical scheme for patients with advanced liver cancer. A Supplementary Table S1 showed the full names of abbreviations.

## Materials and methods

### Callispheres DEB construction

Callispheres DEB (Suzhou Hengrui jialisheng Biotechnology Co., Ltd, Suzhou, China), epirubicin (catalog No. H19990280, Zhejiang Haizheng Pharmaceutical Co., Ltd., 10 mg), sterilized water for injection (catalog No. H41024923, Sinopharm Rongsheng Pharmaceutical Co., Ltd., 2 ml), and iodofol (catalog No. H20143027, Jiangsu Hengrui Pharmaceutical Co., Ltd, 100 ml) were prepared to manufacture drug loaded microspheres used in DEB-TACE. Dissolve the epirubicin with sterile injection water, and the concentration was controlled at 20 mg/ml, then 20-ml syringe was used to extract the dissolved epirubicin for standby. The Callisphere DEB was extracted with 20-ml syringe, and the supernatant was removed after being put for 2 min. Three-way tube was used to connect the syringe with DEB and epirubicin, and epirubicin was injected into the syringe of drug loaded microspheres. The syringe was then shaked once every 5 min for 6 times, so as to make epirubicin completely loaded on DEB. Finally, the contrast agent iodofol was added according to 1.2 times the volume, and then it was shaken well and let stand for 5 min. At this time, the DEB used by DEB-TACE group was well prepare.

### Physicochemical property test of half-time and stability

Callispheres DEB loaded epirubicin, the mixture of iodized oil and epirubicin were administered intraperitoneally in C57BL/6J mouse models with a similar body weight of about 25 g separately (*n* = 2, Shanghai Laboratory Animal Center, Chinese Academy of Sciences, Shanghai, China). The mice were survived during the experiment, and this study was approved by the Ethics Committee of our hospital. Blood samples were taken from the tail vein of the above-injected mice per hour, and epirubicin concentration was detected for the half-time test. The stability of Callispheres DEB was tested by evaluating the diameter alterations in PBS, PBS + 10% serum and PBS+ HepG2 cells, which mimic the blood and tumor environment *in vivo* (20). The sizes of Callispheres DEB were evaluated under scanning electron microscopy (Phenom LE, Phenom Scientific, Netherlands).

### Research subject

Patients with liver cancer (*n* = 96) treated in our hospital were enrolled between March 2017 and March 2019. Inclusion criteria: (1) primary liver cancer diagnosed for the first time; (2) liver cancer was diagnosed according to the criteria in *Asian Pacific Association for the Study of the Liver consensus recommendations on hepatocellular carcinoma* (21); (3) age range: 18–70 years old with an estimated survival time of more than 3 months; (4) received TACE surgery; (5) Barcelona Clinic Liver Cancer (BCLC) staging classification: Phase B or C (22); (6) Eastern Cooperative Oncology Group performance status (ECOG PS): 0–2 (23). Exclusion criteria: (1) previous history of liver surgery; (2) Child-Pugh C cirrhosis (24); (3) severe coagulation dysfunction; (4) hepatic portal vein occlusion or no collateral circulation; (5) combined with active hepatitis; (6) patients received the any treatments, such as c-TACE, ablation, radiotherapy and chemotherapy. Patients were randomly divided into DEB-TACE group (*n* = 48) and c-TACE group (*n* = 48), and treated with DEB-TACE or c-TACE separately. The general data of the two groups were shown in Table 1.

**Table 1:** Baseline data of research subjects.

Baseline information	c-TACE (*n* = 48)	DEB-TACE (*n* = 48)	*P*
Age (year)	50.12 ± 10.23	48.88 ± 10.19	0.553
Gender			
Male	21 (43.75%)	28 (58.33%)	0.196
Female	27 (56.25%)	20 (41.67%)	
BMI (kg/m^2^)	24.41 ± 2.36	24.68 ± 2.45	0.584
HCC	41 (85.42%)	44 (91.67%)	0.336
Hepatitis	37 (77.08%)	30 (62.5%)	0.120
Cirrhosis	35 (72.92%)	30 (62.5%)	0.275
Vascular invasion	34 (70.83%)	26 (54.17%)	0.092
Average diameter (cm)	5.12 ± 1.23	4.78 ± 1.12	0.160
BCLC stages			
Phase B	28 (58.33%)	32 (66.67%)	0.399
Phase C	20 (41.67%)	16 (33.33%)	
Child-Pugh Grading			
Grade A	39 (81.25%)	36 (75.00%)	0.459
Grade B	9 (18.75%)	12 (25.00%)	
ECOG score			
0	23 (47.92%)	25 (52.08%)	0.539
1	16 (33.33%)	17 (35.42%)	
2	9 (18.75%)	6 (12.5%)	

### TACE interventional therapy process

After disinfection and local anesthesia, the femoral artery was punctured according to Seldinger method (25). Then, the celiac trunk and superior mesenteric artery of the patient were imaged to determine the location of liver tumor and understand its blood supply in detail. Then, the microcatheter was superselective intubated to the blood supply site of liver cancer. In c-TACE group, the mixture of iodized oil (10 ml) and epirubicin (20 mg) was used for chemoembolization (26). In DEB-TACE group, the Callispheres DEB loaded epirubicin (80 mg) (27) was extracted with a 1 ml syringe, and the pulse injection method was used for chemoembolization. The effect of vascular embolization was evaluated by angiography after surgery. When the cancer was completely embolized, the operation was completed. After operation, the puncture point was routinely disinfected, pressurized and bandaged, and symptomatic treatment such as anti-inflammatory, liver protection and pain relief were applied. At the same time, patients in DEB-TACE group were treated with oral medication of 400 mg sorafenib (catalog No. H20160201, Bayer pharmaceutical company, Germany) twice a day (27). Withdrawal criteria: progression or deterioration of the disease, serious adverse reactions, decompensation of liver function (grade C).

### Solid tumor efficacy evaluation

The patients were examined by CT/MRI after operation and the survival area of liver tumor was used as the target lesion for efficacy judgment. According to the modified solid tumor efficacy evaluation standard (5), curative effect was divided into complete remission (CR, complete disappearance of target lesion), partial remission (PR, the reduction of target lesion >30%), stable disease (SD, no significant change in target lesion diameter) and progressive disease (PD, the increasing of target lesion >20%), and the objective response rate (ORR) was calculated. ORR = (CR + PR)/total number.

### Detection of tumor markers and indicators of liver function

Fasting venous blood (5 ml) obtained from the patients were centrifuged at 3,000 rpm. The supernatant was then stored at −80°C in a medical refrigerator. Alanine aminotransferase (ALT), aspartate aminotransferase (AST) and total bilirubin (TBIL) in serum were detected by automatic biochemical analyzer (BS-280, Mindray, Shenzhen Mairui Biomedical Electronics Co., Ltd, Shenzhen, China). Besides, serum levels of alpha-fetoprotein (AFP), carbohydrate antigen-199 (CA-199) and vitamin K absence of antagonist-II (PIVKA-II) were detected by automatic chemiluminescence immunoanalyzer (DXL-800, Beckman).

### Safety evaluation

The adverse reaction was evaluated in terms of gastrointestinal reactions (nausea and vomiting), fatigue, impaired liver function, liver pain, fever and so on, the above adverse reactions were counted.

### Follow up

The patients returned to the hospital every 1 month to review liver function, tumor markers and CT/MRI, so as to evaluate the therapeutic effect. Overall survival (OS) and progression free survival (PFS) was defined as duration from the TACE treatment to the death and disease progression, respectively. The follow-up period was 24 months and ended in March 2021.

### Statistical analysis

SPSS 22.0 was adopted for statistical analysis. The quantitative data were described by *n* (%), and *χ*^2^ test was used for inter-group comparison. Besides, the measurement data of normal distribution was described by mean ± SD and *t*-test was employed for inter-group comparison; While the measurement data of non-normal distribution was described by *M* (*P*_25_, *P*_75_), and Mann-Whitney *U* test was employed for inter-group comparison. Meanwhile, the survival analysis was performed by Kaplan-Meier log-rank survival tests. *P *< 0.05 stands for striking difference.

## Results

### Physicochemical properties of callispheres DEB

**Figure 1:**
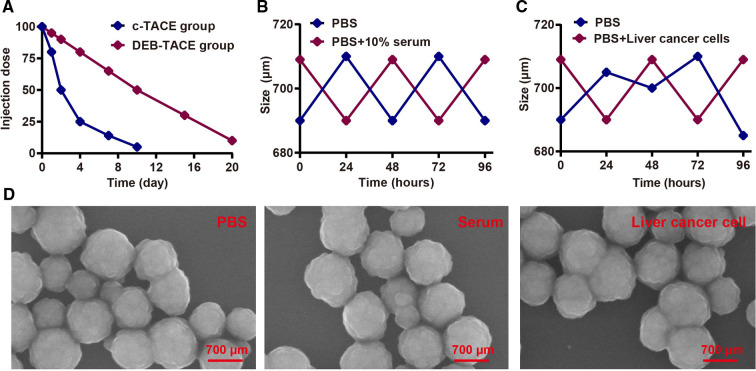
Physicochemical property test of callispheres DEB. (**A**) Half-life period test of Epirubicin in c-TACE and DEB-TACE. (**B**) Stability test of Callispheres DEB in serum. (**C**) Stability test of Callispheres DEB in liver cancer cells. (**D**) Electron microscope structure of Callispheres DEB in PBS, serum and liver cancer cells.

As shown in Figure 1A, epirubicin in DEB-TACE group had a longer half-life than that in c-TACE group (10 vs. 2 days). In terms of stability, Callisphere DEB could effectively maintain its stability in serum and liver cancer cells (Figures 1B,C), and the electron microscope structure. Furthered showed its stability (Figure 1D). As a result, Callisphere DEB could effectively cut off the blood supply of liver cancer cells, thus better inhibit the invasion growth of tumor. In addition, it could reduce the dosage of drugs without affecting the effect.

### Clinical remission after TACE surgery

**Figure 2:**
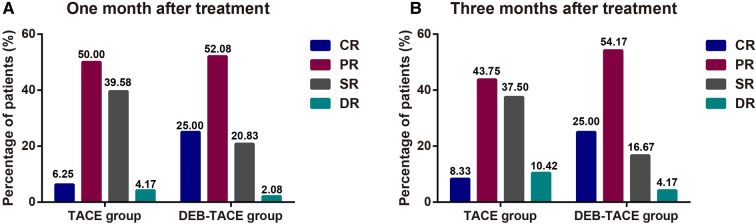
The clinical remission after TACE surgery. The ORR (CR + PR) of patients with liver cancer at 1st (**A**) and 3rd (**B**) month after treatment.

One month after treatment, the ORR of DEB-TACE was higher than that of c-TACE [*P* = 0.030, 95%CI (0.158, 0.924)], suggesting that DEB-TACE could greatly reduce the volume of liver tumors in the short term (Figure 2A). At 3rd month after treatment, the ORR of c-TACE decreased, while it further increased in DEB-TACE group [*P* = 0.005, 95%CI (0.117, 0.702)]. Moreover, CR was higher in DEB-TACE than that in c-TACE [25% vs. 8.33%, *P* = 0.028, 95%CI (0.801, 0.919)] (Figure 2B). The above results showed that DEB-TACE could better inhibit the invasion and growth of tumor, reduce tumor volume and avoid the further progress of liver cancer.

### Changes of serum tumor markers after TACE surgery

One month after treatment, both treatment regimens could reduce the levels of AFP, CA-199, and PIVKA-II, especially in DEB-TACE group (all *P* < 0.05). At 3rd month after treatment, the level of tumor markers in c-TACE group increased without difference when compared with the data at 1 month (all *P* > 0.05), while that in DEB-TACE group continued to decrease (all *P* < 0.05, Table 2). The above results showed that DEB-TACE had better clinical efficacy and more lasting antitumor effect.

**Table 2:** Detection of alpha-fetoprotein (AFP), carbohydrate antigen-199 (CA-199) and vitamin K absence of antagonist-II (PIVKA-II) in serum.

	c-TACE (*n* = 48)	DEB-TACE (*n* = 48)	*P*
AFP (ng/ml)			
Preoperative	624.10 (25.67–3438.4)	588.34 (19.29–3358.12)	0.217
After 1 month	517.13 (18.45–1123.56)*	381.20 (12.13–965.24)*	<0.001
After 3 months	529.35 (19.23–1214.25)*	334.26 (10.36–856.87)*,**	<0.001
CA-199 (U/L)			
Preoperative	80.10 (9.36–453.42)	83.34 (10.29–512.38)	0.110
After 1 month	53.13 (8.45–215.78)*	41.31 (6.13–165.34)*	<0.001
After 3 months	61.35 (9.13–287.98)*	31.26 (6.23–89.34)*,**	<0.001
PIVKA-II (mAU/ml)			
Preoperative	566.34 (47.66–3664.35)	583.75 (40.67–3721.25)	0.543
After 1 month	473.25 (52.39–3178.27)*	383.25 (124.56–2654.62)*	0.002
After 3 months	467.43 (49.65–2945.63)*	176.87 (31.66–829.64)*,**	<0.001

* Compared with preoperative, *P* < 0.05.

** Compared with 1 month, *P *< 0.05.

### Changes of liver function after TACE surgery

One week after treatment, the transaminase and TBIL of both groups greatly increased, and the above indexes increased more significantly in c-TACE group (all *P* < 0.05), considering that it was related to the reduction of blood supply and the liver damage of chemotherapeutic drugs. After 1 month, the liver function of the two groups was relieved, and the above indexes decreased significantly, especially in DEB-TACE group (all *P* < 0.05). After 3 months, the above indexes increased again in both groups, especially in c-TACE group, which were greatly higher than those before treatment (all *P* < 0.05, Table 3). The above results suggested that liver function was damaged greater in c-TACE group than that in DEB-TACE group.

**Table 3:** Comparison of alanine aminotransferase (ALT), aspartate aminotransferase (AST) and total bilirubin (TBIL) in serum between c-TACE group and DEB-TACE group.

	c-TACE (*n* = 48)	DEB-TACE (*n* = 48)	*P*
ALT (U/L)			
Preoperative	80.64 ± 13.03	82.23 ± 12.27	0.540
After 1 week	139.23 ± 23.23*	106.12 ± 19.13*	<0.001
After 1 month	83.02 ± 11.16**	76.59 ± 10.34*,**	0.004
After 3 months	107.58 ± 15.49*,***	83.04 ± 12.71***	<0.001
AST (U/L)			
Preoperative	67.28 ± 10.06	68.14 ± 10.41	0.682
After 1 week	101.24 ± 21.56*	88.12 ± 17.13	0.001
After 1 month	52.23 ± 9.55*,**	45.58 ± 8.13*,**	<0.001
After 3 months	81.19 ± 15.03*,***	65.2 ± 11.42***^#^	<0.001
TBIL (mol/L)			
Preoperative	50.23 ± 7.53	50.06 ± 7.14	0.910
After 1 week	68.34 ± 11.12*	60.13 ± 10.23*	<0.001
After 1 month	51.46 ± 8.39**	47.15 ± 8.02**	0.012
After 3 months	85.37 ± 16.52*,***	71.11 ± 13.34*,***	<0.001

* Compared with preoperative, *P* < 0.05.

** Compared with 1 week, *P *< 0.05.

*** Compared with 1 month, *P *< 0.05.

### Incidence of postoperative adverse events after TACE surgery

**Figure 3:**
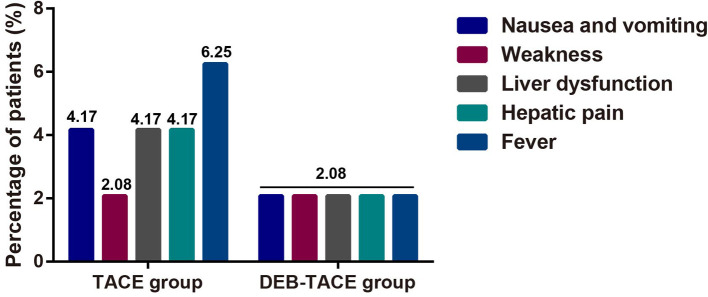
The comparison of postoperative adverse reactions in terms of weakness, liver dysfunction, hepatic pain, fever, nausea and vomiting.

The incidence of adverse reactions such as gastrointestinal reaction fatigue, liver dysfunction, liver pain and fever were greatly lower in DEB-TACE group when compared to the c-TACE group (Figure 3), suggesting that DEB-TACE could significantly reduce the adverse reactions caused by chemotherapeutic drugs and liver tissue ischemia after embolization.

### Survival analysis between DEB-TACE and c-TACE group

**Figure 4:**
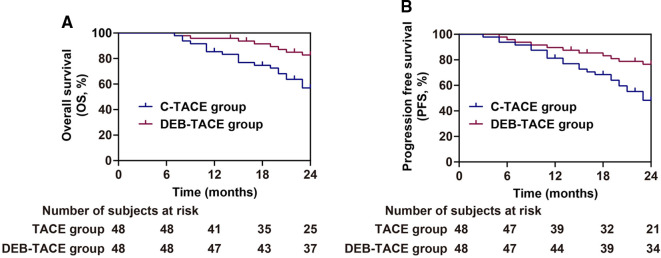
Analysis of prognosis of patients in the c-TACE group and DEB-TACE group. (**A,B**) Overall survival (**A**) and progression free survival (**B**).

After 2 years of follow-up, patients treated with DEB-TACE had higher overall survival rate (*P *= 0.007), and were not easier to relapse and progress (*P *= 0.008) after treatment than those treated with c-TACE (Figure 4). The above results suggested that DEB-TACE could better block the blood supply of liver cancer, inhibit the growth of tumor cells, and effectively avoid tumor recurrence and metastasis.

## Discussion

Primary liver cancer is a common malignant tumor, its incidence ranks fourth, and its mortality ranks second among all malignant tumors in China (28). For these patients who are difficult to be treated surgically, radiotherapy and chemotherapy have become common treatment schemes, which, however, could cause systemic injury (29). The emergence of c-TACE treatment scheme provides a new choice for the treatment of these patients, which can superselect the target artery through microcatheter for arterial embolization to make tumor cells ischemic necrosis, and chemotherapeutic drugs can be loaded around the tumor through iodized oil to improve its efficacy (30). However, iodized oil can not completely block blood vessels because of its poor deposition effect, and is easy to be decomposed and absorbed in the later stage. In addition, the stability of the mixture of iodized oil and chemotherapeutic drugs is poor, which makes chemotherapeutic drugs easy to enter the blood circulation, leading to the increase of toxic effects of drugs (30, 31). Therefore, the development of Callispheres DEB provides a better choice for the therapy of advanced liver cancer.

In our study, the use of Callispheres DEB can help to improve the clinical efficacy, prolong the half-life of chemotherapy drugs. The surface of Callispheres DEB is negatively charged (32). After mixing with chemotherapeutic drugs, the positively charged amino groups in chemotherapeutic drugs can replace sodium ions, and eventually combined stably with hydrogen bond and ion bond forces (33, 34), so as to effectively avoid the loss of chemotherapeutic drugs during transportation. At the same time, after reaching the action target, the chemotherapeutic drugs loaded in Callispheres DEB can be replaced with sodium ions in the blood, so as to continuously release the loaded chemotherapy drugs and maintain the drug concentration around the tumor cells to the greatest extent (35, 36). In addition, Callispheres DEB has non degradability *in vivo*, which helps them to firmly block the target blood vessel after being transported to the target with the blood flow, so as to completely cut off the blood supply of tumor cells (37). In the experiments of physical and chemical properties, it was found that Callispheres DEB could continuously maintain stability in liver cancer cells and significantly prolong the half-life of epirubicin, while iodized oil would be gradually decomposed by liver cancer cells, and epirubicin carried by iodized oil was easier to be metabolized *in vivo*.

After DEB-TACE treatment, the ORR was higher than the c-TACE with lower levels of AFP, CA-199 and PIVKA-II. DEB-TACE has a more thorough embolization effect on blood vessels than c-TACE, and DEB can effectively maintain stability in the blood and continuously release chemotherapeutic drugs. Therefore, DEB-TACE can effectively reduce the tumor volume. Three months after operation, the ORR of c-TACE group decreased compared with that at 1 month after surgery. Similarly, Liu et al. also found that after 3 months of treatment, the ORR of liver cancer patients treated with DEB-TACE combined with sorafenib were higher (38). The reason may be that the embolization effect of iodized oil is poor, resulting in blood vessel recanalization and collateral circulation. In DEB-TACE group, DEB block blood vessels completely, and the use of sorafenib can effectively reduce the expression of antigenic factors in tumor cells, so as to better inhibit the invasion and growth of the tumor and reduce the level of tumor markers.

The study found that the levels of transaminase and TBIL in both groups increased 1 week after operation, especially in c-TACE group. One month after operation, the liver function indexes in DEB-TACE group basically returned to normal, but those in c-TACE group were still high, and increased again at 3 months after operation. The safety of chemotherapeutic drugs is closely concerned in clinical use, especially its liver toxicity. It is suggested that compared with c-TACE, Callispheres DEB can highly selectively kill liver tumor cells and effectively avoid the damage of normal liver cells.

Relevant studies found that the slow-release of DEB could effectively reduce the extravasation of chemotherapeutic drugs and reduce their concentration in the blood, so as to reduce the occurrence of adverse reactions (39, 40). In our study, after DEB-TACE, the incidence of adverse reactions such as gastrointestinal reaction fatigue, liver dysfunction, liver pain and fever were greatly lower than the c-TACE group. Therefore, DEB-TACE combined with sorafenib is safe in the therapy of advanced liver cancer. Thanks to the fact that DEB-TACE combined with sorafenib can better cut off the blood supply of tumor cells and inhibit the formation of neovascularization in tumor, the survival rate is greatly higher than those in c-TACE group. However, several limitations existed in the current study: (1) other liver function indicators, such as indirect bilirubin and albumin would be determined, which are influence factors affecting the treatment of liver cancer; (2) Further randomized controlled trial with larger sample size is needed to validated our result; (3) The power of sample size should be calculated to avoid conclusion bias.

In conclusion, Callispheres DEB can maintain good stability in the blood, effectively prolong the half-life of the chemotherapy drugs, and reduce their extravasation in the blood. Combined with oral medicine of sorafenib, DEB-TACE could further inhibit the formation of neovascularization in tumor, suppress the level of tumor markers, and reduce the adverse reactions. Therefore, DEB-TACE combined with sorafenib can better inhibit tumor invasion and growth and improve the prognosis of patients with unresectable liver cancer.

## Data Availability

The original contributions presented in the study are included in the article/Supplementary Material, further inquiries can be directed to the corresponding author/s.
