# Supplementary material for: Callispheres drug-eluting bead transhepatic artery chemoembolization with oral delivery of sorafenib for the treatment of unresectable liver cancer
Source: Front Surg. 2022 Sep 2;9:981116. doi: 10.3389/fsurg.2022.981116 (PMC9478363; doi:10.3389/fsurg.2022.981116)
Supplement: Supplementary file 1 [file Table_4_v1.docx]

**Supplementary Table 1** The full names of abbreviations

| Abbreviations | Full name |
| --- | --- |
| TACE | Transarterial Chemoembolization |
| c-TACE | Conventional TACE |
| DEB-TACE | Drug-Eluting Bead TACE |
| BCLC | Barcelona Clinic Liver Cancer |
| ECOG PS | Eastern Cooperative Oncology Group Performance Status |
| CR | Complete Remission |
| PR | Partial Remission |
| SD | Stable Disease |
| PD | Progressive Disease |
| ORR | Objective Response Rate |
| ALT | Alanine Aminotransferase |
| AST | Aspartate Aminotransferase |
| TBIL | Total Bilirubin |
| AFP | Alpha-Fetoprotein |
| CA-199 | Carbohydrate Antigen-199 |
| PIVKA-Ⅱ | Vitamin K Absence Of Antagonist-Ⅱ |
| OS | Overall Survival |
| PFS | Progression Free Survival |
